# Evaluation of intrapulmonary arteriovenous anastomoses before and after oxygen supplementation, using transthoracic agitated saline contrast echocardiography in rescued Korean raccoon dogs

**DOI:** 10.3389/fvets.2024.1362363

**Published:** 2024-08-08

**Authors:** Chang-eun Lee, Myeongsu Kim, Jae-Ik Han, Kichang Lee, Hakyoung Yoon

**Affiliations:** ^1^Department of Veterinary Medical Imaging, College of Veterinary Medicine, Jeonbuk National University, Iksan-si, Republic of Korea; ^2^Laboratory of Wildlife Medicine, College of Veterinary Medicine, Jeonbuk National University, Iksan-si, Republic of Korea; ^3^Jeonbuk Wildlife Center, Jeonbuk National University, Iksan-si, Republic of Korea; ^4^Biosafety Research Institute and College of Veterinary Medicine, Jeonbuk National University, Iksan-si, Republic of Korea

**Keywords:** IPAVA, bubble study, hyperoxia, right to left shunt, transpulmonary shunt

## Abstract

**Introduction:**

Intrapulmonary arteriovenous anastomoses (IPAVAs) are defined as relatively large, dynamic shunt vessels that connect the pulmonary arterial and venous systems, thereby bypassing the pulmonary capillary system. IPAVAs lower elevated pulmonary arterial pressure; however, the presence of the shunt can result in impaired pulmonary gas exchange and paradoxical embolism. Furthermore, the prevalence and effects of IPAVAs in raccoon dogs remain unknown. This study aimed to determine the prevalence of IPAVA among rescued Korean raccoon dogs and evaluate the changes in IPAVA following oxygen supplementation.

**Methods:**

Nineteen raccoon dogs rescued by the Jeonbuk Wildlife Centre between August 2022 and December 2023 were subjected to echocardiography. Sixteen healthy and three abnormal raccoon dogs were subjected to transthoracic agitated saline contrast echocardiography (bubble study) based on the echocardiography results. IPAVA was considered to be present if the left heart contrast was visualised after four cardiac cycles following the visualisation of the first right heart contrast. Bubble scores (BS0–5) were assigned based on the maximum number of microbubbles observed in the left ventricular lumen per frame of the ultrasound image. BS was assigned before and after supplementation with 100% oxygen for 5 min.

**Results:**

IPAVA was detected in 12 of the 16 healthy raccoon dogs at rest (75%). The BS of the 15 IPAVA-positive raccoon dogs ranged from 1 to 4 points (BS1, 1; BS2, 4; BS3, 8; and BS4, 2). Blood flow through the IPAVA (Q_IPAVA_) was reduced or absent in the 15 IPAVA-positive raccoon dogs after supplementation with 100% oxygen (BS0, 11; BS2, 4). Moreover, BS of the IPAVA showed a significant correlation with the cardiac output per body weight (BW).

**Conclusion:**

The prevalence of IPAVA in healthy raccoon dogs at rest was 75%. Adequate oxygen supplementation was found to be effective in reducing Q_IPAVA_, which may help prevent potential negative factors such as pulmonary gas exchange impairments and paradoxical embolism that can occur with IPAVA.

## 1 Introduction

Intrapulmonary arteriovenous anastomoses (IPAVAs) are shunt vessels larger than the pulmonary capillaries that connect the pulmonary arterial system to the pulmonary venous system, thereby bypassing the pulmonary capillaries (1–4). The presence of these unusual blood vessels in humans was first suggested ~100 years ago (5); however, the pathological or physiological significance of these structures remains controversial and has not been fully elucidated (2, 6).

A characteristic feature of IPAVA is that it is a dynamic vessel that can open and close under the influence of specific factors. Oxygen concentration is one such factor. Blood flow through IPAVA (Q_IPAVA_) in humans may increase under conditions of hypoxia (7–10) and decrease under conditions of hyperoxia (7, 11). A pronounced increase in Q_IPAVA_ during exercise (12–15) has also been observed in humans, dogs, and horses. Q_IPAVA_ increases the concentration of catecholamines, such as epinephrine, dopamine, and dobutamine, in humans (16–18). Q_IPAVA_ in humans has been shown to decrease in the lateral recumbent position compared with that in the upright position (19). Thus, posture, as well as endogenous and exogenous neurotransmitters, affect IPAVA. IPAVA is more prevalent during the foetal and neonatal periods than during adulthood in sheep (20). Similarly, it is less prevalent among older adults in humans (12).

It is unclear whether IPAVA is a physiological or pathological variation, and its clinical implications and relevance remain unknown (21). IPAVA may be a remnant of non-degenerating foetal blood vessels in the lungs. These vessels may originally be present during the foetal period when pulmonary gas exchange is not required and gradually disappear after birth (4, 20, 22). Pulmonary hypertension (PH) is less prevalent among patients with IPAVA, suggesting that IPAVA may play a physiological regulatory role by minimising the damaging effects of increased pressure and reducing right ventricular (RV) afterload (12, 22, 23). However, the presence of the IPAVA is associated with several complications. As Q_IPAVA_ bypasses pulmonary capillaries, it frees these vessels from their role in pulmonary gas exchange and biological filtration. Studies conducted using microspheres have shown that 1.4 ± 0.8% of the cardiac output is shunted in dogs with IPAVA. Studies conducted using 99mTC-MAA have shown that >5% of the cardiac output is shunted in humans (8, 13). As even 2% of shunting results in a decrease in the alveolar-to-arterial oxygen difference, pulmonary gas exchange can be impaired by the increase in Q_IPAVA_ (2). Paradoxical embolism is another clinical manifestation of IPAVA wherein large substances filtered out by the pulmonary capillaries pass into the systemic circulation as the diameter of IPAVA (>25 or 50 μm) is much larger than that of the pulmonary capillaries (7–10 μm) (3, 13, 24, 25). Emboli of <100 μm in size can lead to cerebral ischaemia and infarction in rats. Paradoxical emboli have been known to pass through a pulmonary arteriovenous malformation (PAVM), which is similar to IPAVA, resulting in ischaemic stroke in humans (26, 27). Thus, an increase in the Q_IPAVA_ may increase the risk of pulmonary gas exchange impairment and paradoxical embolism (8, 13, 14, 28, 29). Paradoxical embolism induced by IPAVA has been observed in several clinically important conditions in humans, such as stroke, transient ischaemic attack, migraine, and gas embolism during scuba diving (26, 29–31).

Several methods, such as microspheres, macroaggregated albumin (MAA), and agitated saline contrast using microbubbles (bubble study), have been used for the detection of IPAVA. These methods have several advantages and disadvantages. However, these methods use substances that are normally filtered or removed from the pulmonary capillaries to identify a right-to-left shunt. Microspheres are composed of polymer or glass. Consequently, these structures are not easily deformed and maintain a constant size (e.g., 25 and 50 μm). These properties have been used to determine the diameter of the vessel through which they pass. However, *in vivo* experiments are not feasible as biodegradable products are not yet commercially available. Consequently, microspheres are mainly used in *ex vivo* experiments, such as isolated lungs (1, 3, 13). Technetium-99 m labelled MAA (99mTc-MAA), an MAA tagged with radioactive material, has also been used to detect IPAVAs. Owing to its biodegradability, 99mTc-MAA is safe for use in humans. Moreover, it enables quantification of the shunt degree. However, 99mTc-MAA requires advanced equipment, such as a gamma camera, and the quantification of the shunt may be somewhat overestimated, as free 99mTC isolated from MAA passes through the pulmonary capillaries (1, 8, 28). Bubble study is performed by mixing normal saline with air to create microbubbles and injecting the mixed saline solution intravascularly. The presence of the bubbles is confirmed via echocardiography. This method does not quantify the extent of the shunt as well as microspheres and 99mTc-MAA. In addition, the resulting microbubbles are unevenly sized and fragile. However, bubble study has enabled a qualitative assessment of Q_IPAVA_ and can be used to distinguish whether the shunt is intracardiac or extracardiac. Furthermore, it uses readily available materials, is safe for living organisms, and can be used on unanaesthetised animals. Thus, bubble study is an inexpensive, safe, and convenient method (1, 32, 33), and its use in research and clinical practise has increased in recent years (34–36).

IPAVAs have primarily been studied in humans (37), dogs, sheep, horses, and rats. However, most of these studies were *ex vivo* studies that used isolated lungs (10, 13, 15, 20). To the best of our knowledge, no previous study has focused on the prevalence of IPAVA in healthy animals at rest or whether hyperoxia reduces Q_IPAVA_ in animals, as it does in humans. Notably, IPAVAs have not been studied in raccoon dogs. Therefore, the present study aimed to determine the prevalence of IPAVA in healthy raccoon dogs at rest and evaluate the changes in IPAVA following supplementation with 100% oxygen.

## 2 Materials and methods

### 2.1 Animals

This prospective study included 19 wild Korean raccoon dogs (scientific name: *Nyctereutes procyonoides koreensis*) that were rescued or captured by the Jeonbuk Wildlife Centre between August 2022 and December 2023. The 19 raccoon dogs comprised 13 males and 6 females, with a mean (± standard deviation) weight of 3.5 ± 1.25 kg. All raccoon dogs underwent simple health examinations, such as physical examinations and blood tests. Thoracic radiography and echocardiography were performed subsequently to confirm the presence of cardiac disease. A bubble study was conducted while breathing room air [true inspired oxygen fraction (FiO_2_): 0.21] and echocardiography was performed to confirm the presence and extent of IPAVA. The IPAVA-positive raccoon dogs were administered 100% oxygen via a mask at a rate of 5 L/min for 5 min. The FiO_2_ was assumed to range from 0.5 to 0.7, as reported in previous similar studies, in accordance with the guidelines of the American Association of Respiratory Care (19, 38).

This study was approved by the Institutional Animal Care and Use Committee of the Jeonbuk National University (Approval No. JBNUNON2022-081-002).

### 2.2 Echocardiography and cardiac indicators

All ultrasound examinations were performed using Aplio i800 (Canon Medical Systems, Tokyo, Japan). A cardiac sector probe, PST-50BT (frequency, 3.0–8.2 MHz), was used to perform echocardiography. Raccoon dogs were placed in the left and right lateral recumbent positions and examined through the left (LPS) and right parasternal sternal (RPS) windows. Any abnormalities in morphology, movement, and blood flow through the heart and valves were evaluated to differentiate congenital cardiac diseases from acquired cardiac diseases.

The cardiac parameters were measured in each raccoon dog. The heart rate was determined using electrocardiography. Tricuspid regurgitation (TR) velocity was measured using continuous wave (CW). Doppler to assess the pulmonary artery systolic pressure and PH. The stroke volume was calculated using the Teichholz formula with the systolic and diastolic diameters of the left ventricular lumen measured along the long or short axis of the RPS view (39). Cardiac output was calculated as the product of stroke volume and heart rate. Cardiac output was divided by the body surface area (BSA) and body weight (BW) to obtain the cardiac output index and cardiac output per BW, respectively. Other basic echocardiography indicators, such as the diameters of the left atrium, aorta, and main pulmonary artery; the thickness of the left ventricular wall; the e-peak and a-peak of the mitral valve flow; and the presence of regurgitation were also measured.

### 2.3 Bubble study (agitated saline contrast echocardiography)

The mixed saline solution, comprising 9 mL normal saline (NS), 0.5 mL room air, and 0.5 mL blood, was pushed back and forth between two 10 mL syringes connected perpendicularly to a three-way stopcock until the large visible air bubbles disappeared and the mixture became homogeneously turbid. Blood was added to lower the surface tension of the microbubbles and increase cohesion, thereby increasing the stability and longevity (34). Each raccoon dog was placed in the left lateral recumbent position and 2 mL of the agitated mixed saline solution was administered through a 23G intravascular catheter placed in the cephalic vein. Bubble contrast images of the right and left hearts were acquired immediately after the infusion using LPS apical four-chamber echocardiography. Imaging was continued until all intracardiac bubbles disappeared.

**Figure 1:**
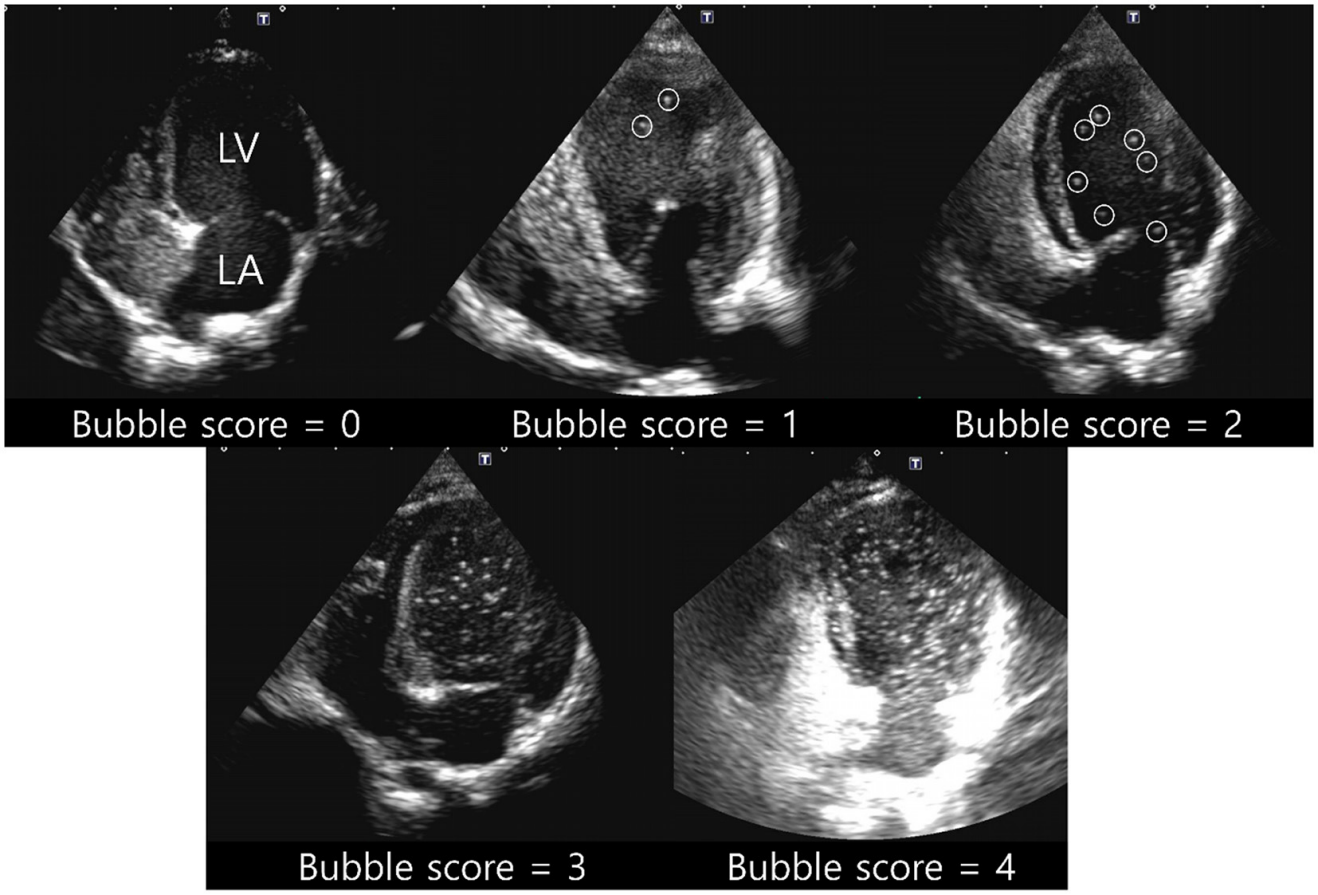
Representative pictures for bubble score (BS) of 0–4. BS0, 0 microbubbles; BS1, 1–3 microbubbles; BS2, 4–12 microbubbles; BS3, >12 microbubbles; BS4, >12 microbubbles heterogeneously distributed throughout the left ventricle; BS5, >12 microbubbles homogeneously distributed throughout the left ventricle. Circles of BS1 and BS2 indicate each bubble. BS5 was not observed in raccoon dogs. LA, left atrium; LV, left ventricle.

RadiAnt DICOM Viewer (Medixant, Poznan, Poland) was used to evaluate the contrast pattern in the acquired images. The emergence of the left heart bubble contrast within four cardiac cycles of the appearance of the first right heart bubble contrast was evaluated. An intracardiac shunt was considered to be present if the left heart bubble appeared within four cardiac cycles. In contrast, an extracardiac shunt, including IPAVA, was considered to be present if the left heart bubble appeared after four cardiac cycles. The criteria for categorising cardiac cycles for intracardiac and extracardiac shunts in the bubble study were based on those recently proposed in another human study (40). Bubble scores were assigned based on the maximum number of bubbles observed per frame within the left ventricle (LV). The bubble scores ranged from 0 to 5 points and this system has been used in many bubble studies (Figure 1): Bubble score (BS) 0, 0 microbubbles; BS1, 1–3 microbubbles; BS2, 4–12 microbubbles; BS3, >12 microbubbles; BS4, >12 microbubbles heterogeneously distributed throughout the left ventricle; and BS5, >12 microbubbles homogeneously distributed throughout the left ventricle (19, 41). Cases with BS0 and BS1–5 were classified as IPAVA negative and positive, respectively.

### 2.4 Statistical analysis

The Wilcoxon signed-rank test was used to evaluate significant differences in the BS and the maximum number of bubbles within the LV of IPAVA-positive raccoon dogs before and after supplementation with oxygen. Spearman's rank correlation test was used to evaluate the correlation between BS and the cardiac indicators. Raccoon dogs with cardiac disease were excluded owing to the presence of several variables that could be involved, including changes in haemodynamics. Only cardiac indicators from healthy raccoon dogs were used for the correlation analysis. Intraclass correlation coefficients (ICCs) with 95% confidence intervals (CIs) were calculated to determine the intra- and inter-observer reliability for the maximum number of bubbles in the left ventricle (excluding BS0). One author (Observer A) measured the items twice to determine the intra-observer reliability, whereas five observers, including the author (Observers A–E), measured the items to determine the inter-rater reliability. Statistical significance was set at *p-*values <0.05^*^ or <0.001^**^. All statistical tests were performed using IBM SPSS Statistics (version 20.0; IBM Corp., Armonk, NY, USA).

## 3 Results

### 3.1 Echocardiography

**Figure 2:**
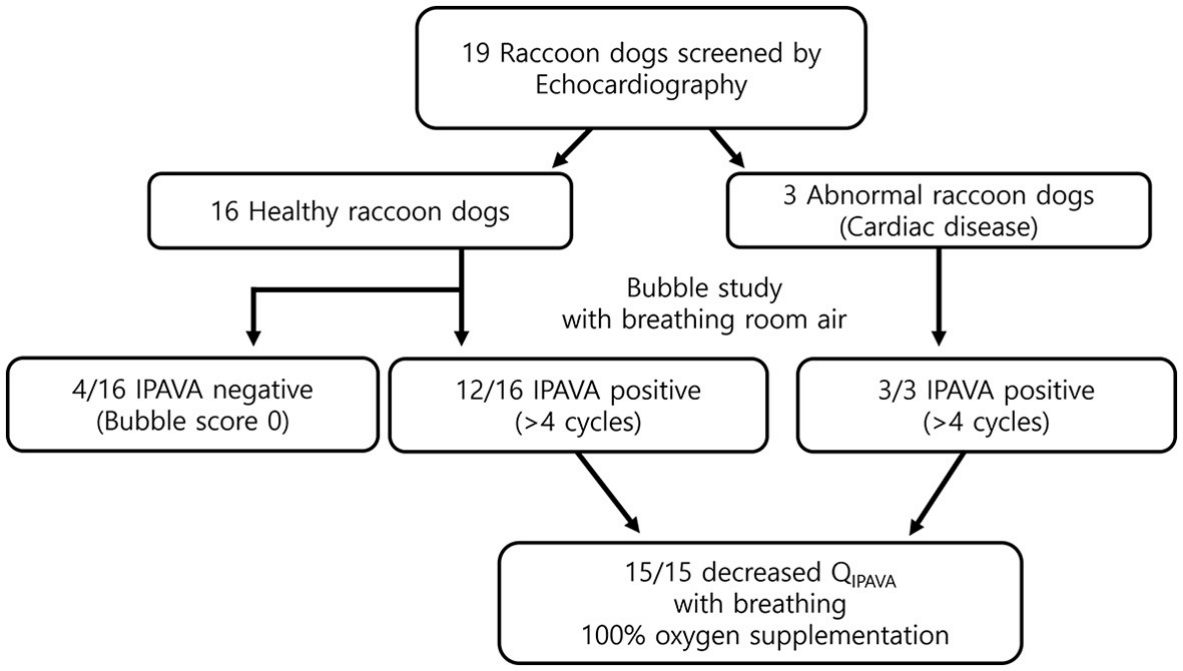
Flow chart of the study. Twelve healthy and three abnormal animals (i.e., with cardiac disease) were identified through echocardiography. The prevalence of IPAVA in healthy raccoon dogs was 75% (9/12). A decrease in the bubble score was observed in all IPAVA-positive animals (12/12) following supplementation with 100% oxygen for 5 min.

All 19 raccoon dogs underwent echocardiography. Sixteen healthy and three abnormal raccoon dogs with cardiac disease were identified (Figure 2). Mitral valve insufficiency was detected in two of the three abnormal raccoon dogs, whereas a ventricular septal defect (VSD) and pulmonary stenosis (PS) were detected in the third raccoon dog. All 16 healthy raccoon dogs had trivial to no TR.

### 3.2 IPAVA and bubble score

The appearance of left heart contrast after four cardiac cycles following the visualisation of the first right heart contrast was observed in 12 of the 16 healthy raccoon dogs (Figure 2). After excluding the four IPAVA-negative (BS0) raccoon dogs, one, three, seven, and one raccoon dog(s) had BS1, BS2, BS3, and BS4, respectively (Table 1). Thus, the prevalence of IPAVA in healthy raccoon dogs at rest was ~75% (12/16). The three abnormal raccoon dogs with cardiac disease were IPAVA-positive, with BS2, 3, and 4 (Table 1). BS5 was not observed in any of the bubble studies. The left heart contrast emerged after four cardiac cycles following the appearance of the first right heart contrast.

**Table 1:** Bubble scores and maximum numbers of bubbles in the IPAVA-positive raccoon dogs breathing room air and 100% oxygen.

**Animal**	**Echocardiography**	**Bubble score (max. number of bubbles)**	
		**Room air**	**100% oxygen**
1	Normal (healthy)	3 (25)	2 (10)
2		2 (6)	0 (0)
3		3 (25)	2 (7)
4		1 (3)	0 (0)
5		3 (20)	0 (0)
6		2 (5)	0 (0)
7		4 (55)	2 (8)
8		3 (25)	0 (0)
9		2 (8)	0 (0)
10		3 (14)	0 (0)
11		3 (13)	0 (0)
12		3 (14)	0 (0)
13	Cardiac disease	4 (67)	2 (6)
14		2 (7)	0 (0)
15		3 (21)	0 (0)

### 3.3 Oxygen supplementation

**Figure 3:**
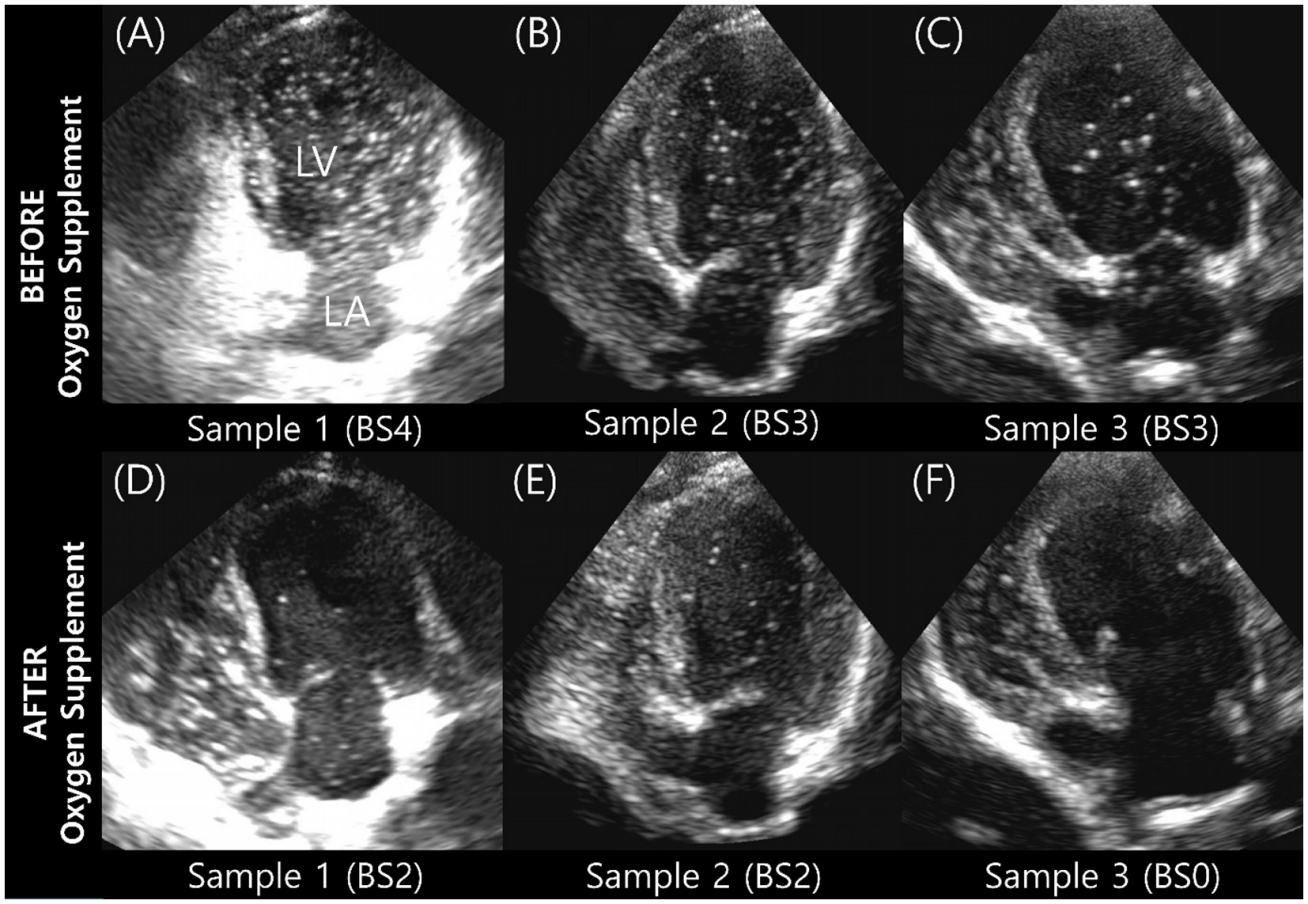
Images of three subjects acquired during the bubble study before and after oxygen supplementation. **(A–C)** Are pre-oxygen supplementation images. **(D–F)** Are the changes in each **(A–C)** after supplementation with 100% oxygen for 5 min. Bubble contrast within the left ventricle was clearly reduced in all subjects, indicating that oxygenation reduces Q_IPAVA_. BS, bubble score; LA, left atrium; LV, left ventricle.

**Figure 4:**
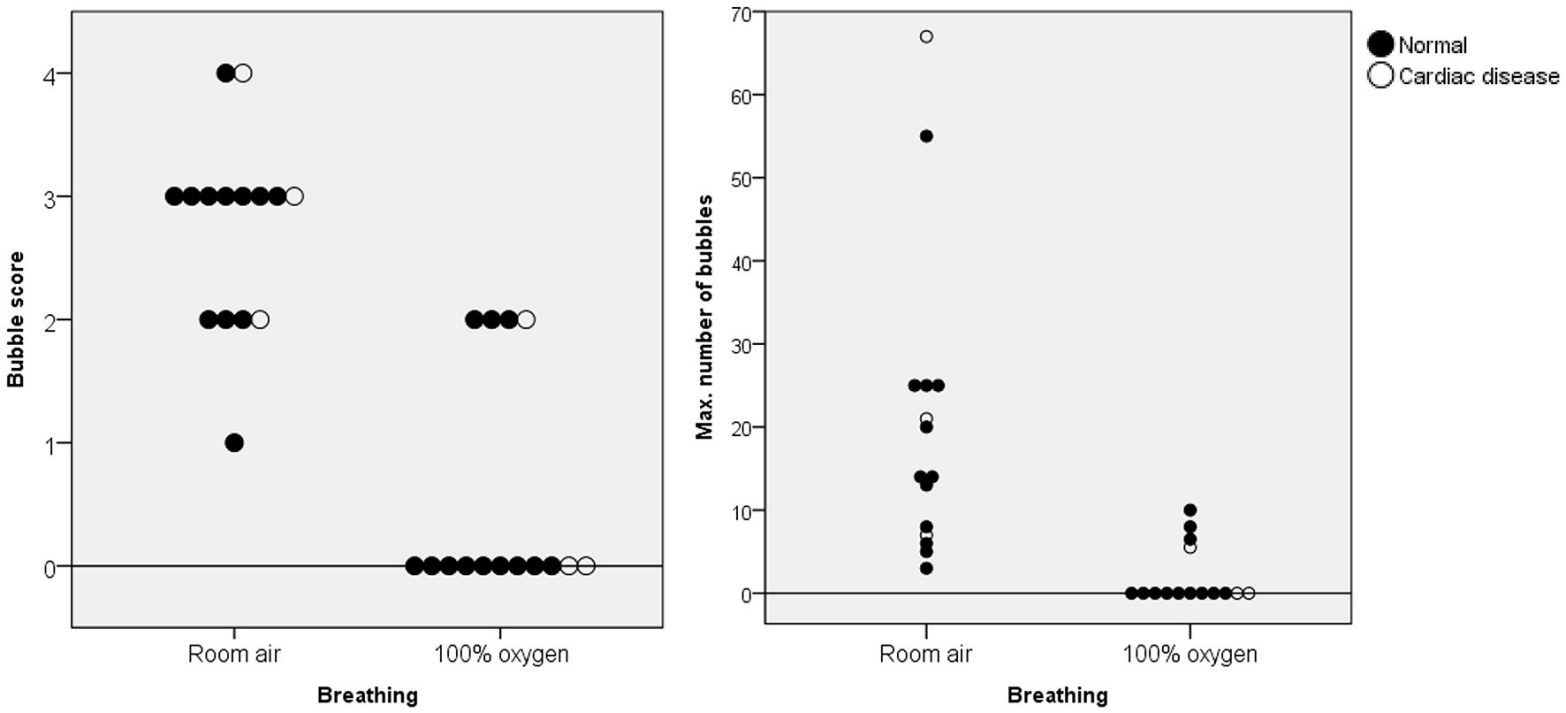
Bubble scores and maximum number of bubbles in the left ventricle for each animal breathing room air and 100% oxygen for 5 min. The black data points indicate normal cardiac function, whereas the white data points indicate abnormal cardiac function. A significant decrease in these figures was observed in all animals after supplementation with 100% oxygen.

The BS and maximum number of bubbles in the LV decreased 5 min after supplementation with 100% oxygen in all 15 IPAVA-positive raccoon dogs (Table 1, Figure 3). A decrease in Q_IPAVA_ was observed after sufficient oxygenation in all 15 IPAVA-positive raccoon dogs (Figure 4). Before oxygenation, there were one, four, eight, and two cases with BS1, BS2, BS3, and BS4, respectively. After oxygenation, there were 11 and four cases with BS0 and BS2, respectively. Thus, a significant reduction in BS and maximum bubble count was observed after oxygenation (*p* = 0.001; Figure 4).

### 3.4 Correlation between the bubble score and the cardiac indicators

Table 2 presents the cardiac indicators of the 16 healthy raccoon dogs. No clear correlations were observed between BS and heart rate, stroke volume, cardiac output, or cardiac output index (*p* > *0*.05); however, BS was correlated with cardiac output per BW. The *p*-value was 0.036 (*p* < 0.05^*^), and Spearman's rank correlation coefficient (ρ) was 0.526, indicating a moderate relationship.

**Table 2:** Bubble scores, basic information, and cardiac indicators of healthy raccoon dogs breathing room air.

**Animal**	**Sex**	**Bubble score (maximum number of bubbles)**	**Weight (kg)**	**Heart rate (beats/min)**	**Stroke volume (mL)**	**Cardiac output (L/min)**	**Cardiac output index (L/min/m^2^)**	Cardiac output per BW^*^(L/min/kg)
1	F	0 (0)	4.3	92	11.93	1.1	4.1	0.255
2	M	3 (25)	3.4	126	12.14	1.5	6.7	0.450
3	M	2 (6)	3.4	159	9.70	1.5	6.8	0.454
4	M	3 (25)	3.9	112	14.19	1.6	6.3	0.407
5	F	0 (0)	4.5	168	3.61	0.6	2.2	0.135
6	M	1 (3)	4.8	65	12.16	0.8	2.8	0.165
7	M	3 (20)	3.9	174	4.67	0.8	3.2	0.208
8	M	0 (0)	3.0	93	7.21	0.7	3.2	0.224
9	M	2 (5)	3.5	148	10.26	1.5	6.5	0.434
10	M	4 (55)	1.4	120	5.35	0.6	5.1	0.459
11	F	3 (25)	1.0	176	2.08	0.4	3.6	0.367
12	F	2 (8)	5.2	122	6.14	0.7	2.5	0.144
13	M	3 (14)	4.1	109	6.78	0.7	2.9	0.180
14	F	3 (13)	3.1	97	9.30	0.9	4.2	0.291
15	F	0 (0)	4.6	78	12.19	1.0	3.4	0.207
16	M	3 (14)	3.4	131	7.71	1.0	4.4	0.297
*p*-value		-.	-.	0.227	0.366	0.973	0.109	0.036^*^

The numbering of animals is not the same as that in Table 1.

^*^Significant correlation with bubble score (p <0.05^*^, ρ = 0.526).

### 3.5 Intra- and inter-observer reliability

Table 3 presents the intra- and inter-observer reliability for measuring the maximum number of bubbles observed in the LV during the bubble study. The intra-observer reliability, assessed based on the two measurements taken by Observer A (first author), was very high, with an ICC of 0.994 (95% CI, 0.986–0.998). The inter-observer reliability, assessed based on the measurements taken by the five observers (Observers A–E), was also very high, with an ICC of 0.995 (95% CI, 0.989–0.998).

**Table 3:** Intra-observer and inter-observer reliability for the maximum number of bubbles in the left ventricle using ICC and the 95% CI.

**Parameter**		**Mean ±SD (number of bubbles)**	**ICC**	**95% CI**	***p-*value**
Intra-observer (observer A)			0.994	0.986–0.998	*p* < 0.001^**^
	First	17.89 ± 17.61			
	Second	17.37 ± 16.34			
Inter-observer			0.995	0.989–0.998	*p* < 0.001^**^
	Observer A	17.63 ± 16.93			
	Observer B	20.21 ± 19.14			
	Observer C	18.26 ± 16.09			
	Observer D	20.21 ± 19.50			
	Observer E	19.53 ± 17.15			

CI, confidence interval; ICC, intra-class correlation coefficient; SD, standard deviation.

Experimental values were considered significant at p <0.001^**^.

## 4 Discussion

This study investigated the prevalence of IPAVA and changes in IPAVA after oxygenation supplementation in 19 wild raccoon dogs using a bubble study and echocardiography.

### 4.1 Bubble study: left heart bubble contrast indicates IPAVA after four cardiac cycles

The detection of IPAVA has become crucial owing to its clinical importance. A bubble study was used for the detection of IPAVA in the present study. Bubble studies are performed by creating microbubbles using agitated saline and are widely used for various purposes. The detection of right-to-left shunts of blood flow is its most notable purpose (25, 30, 34). Intravenously injected microbubbles are filtered or removed as they pass through the pulmonary capillaries. Consequently, they do not reach the left heart in normal cases (24). However, the presence of a right-to-left shunt pathway that bypasses the capillaries in the lungs, which serve as philtres, facilitates the passage of the microbubbles to the left side of the heart. This right-to-left shunt can be categorised as intracardiac or extracardiac. Extracardiac shunts are primarily observed in the lungs and include IPAVAs and pulmonary arteriovenous malformations (PAVMs). Thus, the left heart bubble contrast observed in the present bubble study could also be indicative of an intracardiac shunt or PAVM, in addition to IPAVA. However, intracardiac shunt and PAVM were considered unlikely for several reasons.

The left heart bubble contrast was visualised after four cardiac cycles following the visualisation of the first right heart contrast in all cases in the present study. Thus, the presence of an intracardiac right-to-left shunt was ruled out (40). Intracardiac shunts include patent foramen ovale (PFO; observed in ~25–40% of humans) and other congenital septal defects, such as atrial septal defect (ASD) and ventricular septal defect (VSD). ASD and VSD can cause right-to-left shunts by increasing right heart pressure (19). Intra- and extracardiac right-to-left shunts can be differentiated using bubble studies. An intracardiac shunt is considered to be present if the left heart contrast is visualised within four cardiac cycles after the first visualisation of the right heart contrast. An extracardiac shunt is considered to be present if the left heart contrast is visualised after four cardiac cycles (40). This may be attributed to the fact that the microbubbles that appear in the right heart reach the left heart relatively late if they travel through the lungs. VSD was detected in one raccoon dog via echocardiography just prior to the bubble study in the present study; however, the presence of an intracardiac shunt was unlikely as the left heart contrast did not appear within four cardiac cycles. Thus, bubble study can be used to distinguish extracardiac right-to-left shunts, including IPAVA, from intracardiac right-to-left shunts by evaluating how many cardiac cycles later the left heart bubble contrast is visualised after the first visualisation of the right heart contrast.

In addition to IPAVA, extracardiac shunts also include PAVMs, which are similar to IPAVA. PAVMs can be confused with IPAVA as the left heart contrast is visualised after four cardiac cycles after the first visualisation of the right heart contrast in the bubble study in patients with PAVM as well (4, 27, 40). However, unlike IPAVA, which may be a remnant of a foetal or neonatal structure, PAVM can appear in conjunction with genetic disorders, such as hereditary haemorrhagic telangiectasia, or be acquired due to trauma, infection, or iatrogenic causes (4, 20, 22). An accurate diagnosis of PAVM can be obtained using computed tomography angiography, especially in cases where in a large volume of shunting is observed, as the diameter of PAVM in humans is 1–5 cm, which is much higher than that of IPAVA (4, 27). However, unlike IPAVA, which exhibits dynamic opening and closing, PAVM is static and is assumed to remain open, thereby facilitating a constant flow of blood. Thus, the bubble score is not expected to change even under special conditions, such as oxygen supplementation, in cases with PAVM (1). A reduction in BS was observed in all IPAVA-positive subjects after oxygen supplementation in the present study, which was indicative of IPAVA rather than PAVM. Therefore, changes in the BS through bubble studies before and after oxygen supplementation can be used to differentiate IPAVA from PAVM.

In addition to a right-to-left shunt that bypasses the pulmonary capillaries, the passage of the microbubbles through the dilated pulmonary capillaries can also lead to the visualisation of LV bubble contrast. However, this seems unlikely, given the vessel diameters and bubble sizes reported in previous studies. Microbubbles in agitated saline are <30 μm in size; however, the sizes are irregular (42). In contrast, the diameter of pulmonary capillaries ranges from 7 to 10 μm in size in dogs and does not exceed 13–15 μm even under non-physiological pressure (43, 44). Therefore, the passage of microbubbles through the dilated pulmonary capillaries seems to be impossible. The diameter of IPAVA was found to range from 25 to 50 μm in an experiment using microspheres. Microbubbles can pass through this site easily (3, 13). These findings suggest that the microbubbles created in the bubble study cannot pass through normal pulmonary capillaries and that IPAVA is likely to be the cause if microbubbles appear dynamically in the left ventricle after four cardiac cycles following the visualisation of the right heart contrast.

The left heart contrast observed in raccoon dogs in the bubble study was considered to be indicative of IPAVA and not of an intracardiac shunt, PAVM, or dilated pulmonary vessels, as the left heart contrast appeared within four cardiac cycles after the first visualisation of the right heart contrast and showed dynamic changes depending on supplementation with oxygen. This bubble study method can be easily and conveniently used in the future to detect IPAVA in various species and predict the risks associated with right to left shunt.

### 4.2 IPAVA prevalence in healthy raccoon dogs at rest

The prevalence of IPAVA was ~75% (12/16) among the healthy raccoon dogs at rest in the present study, which is much higher than the previously reported prevalence of 28% in humans (19). Although studies related to IPAVA have been conducted in other animal species, the prevalence of IPAVA in the resting state remains underexplored (10, 13, 15). Studies on horses and dogs have addressed the changes in IPAVA before and after exercise, and no changes in Q_IPAVA_ were observed in any of the subject before exercise (13, 15). Changes in Q_IPAVA_ were observed during normoxia and hypoxia in a previous study involving rats, and Q_IPAVA_ was not observed during normoxia in any of the rats (10). This suggests that the prevalence of IPAVA in healthy raccoon dogs at rest may be higher than that in other animal species. However, bubble studies were not used in the studies involving dogs and rats, and the sample size was <10 for all three species; therefore, direct comparison was difficult (10, 13, 15).

Several factors, such as catecholamine secretion, the lateral recumbent position during testing, and age, may have contributed to the increased prevalence of IPAVA in healthy raccoon dogs at rest observed in the present study. The raccoon dogs included in the present study were wild animals. Moreover, they were not sedated or anaesthetised. Thus, the possibility of elevated catecholamine levels in the body cannot be ruled out even in a resting (apparently stable) state without exercise. Intravenous administration of catecholamines, such as epinephrine, dopamine, and dobutamine, has been shown to increases Q_IPAVA_ by elevating the pulmonary artery systolic pressure (PASP) and/or cardiac output (16, 17). However, the catecholamine levels were not measured in the raccoon dogs in the present study; therefore, these causes cannot be substantiated. The lateral recumbent position assumed during bubble studies and echocardiograms may have also affected the IPAVA. Left heart contrast enhancement observed in human in the supine position disappears in the upright position, which may be attributed to altered pulmonary blood flow distribution due to a change in gravitational action on pulmonary blood flow (14, 19). Age is another factor that may have affected the IPAVA. The prevalence of IPAVA was found to be higher in foetal and neonatal lambs than that in adult lambs in a previous study (20). Similarly, the prevalence of IPAVA was found to be higher in younger individuals than in older individuals in human studies (12). The raccoon dogs included in this study were wild animal whose ages could not be estimated accurately; however, there were some young raccoon dogs weighing <1 kg. If a majority of the rescued raccoon dogs were young, this could explain the high prevalence of IPAVA. Further studies on the prevalence of IPAVA in raccoon dogs according to age, such as comparisons of immature and mature dogs, must be conducted in the future.

Other technical influences may also have had some effect on the bubble study. Unlike previous bubble studies, a small amount of blood from each raccoon dog was mixed in the solution during the preparation of the agitated saline contrast in the present study. This may have increased the stability and longevity of the microbubbles and facilitated their delivery to the left heart (34). Furthermore, differences in the ultrasound machine equipment and the manufacturer, product, and setting values of the probes used may have resulted in better detection of the microbubbles in the heart. Therefore, further studies must be conducted to compare the results of the bubble study of various microbubble manufacturing protocols and ultrasound machines.

Lastly, the high prevalence of IPAVA in raccoon dogs observed in the present study may be attributed to the species-specific nature of raccoon dogs. The prevalence of IPAVA may be higher among raccoon dogs compared with other species of animals or these structures may open more easily in the resting state. Thus, the prevalence of IPAVA may vary between species. Identifying the causative factors would provide insights into IPAVA.

### 4.3 IPAVA in animals

IPAVAs have been studied extensively in humans. These structures are also observed in animals, and studies have been conducted in various species, such as dogs, horses, and rats (1, 10, 13, 15, 37). However, the experimental methods used in the present study involving raccoon dogs differs from those of previous animal studies. First, *in vivo* and *ex vivo* experiments (living dogs and isolated lungs) were conducted in previous studies involving dogs, and the presence or absence of microspheres in blood or tissue was confirmed before and after exercise through commercial microsphere injection rather than through the use of bubble study. Moreover, the primary objective of a previous canine study was to determine the prevalence of exercise-induced IPAVA (13). Changes in IPAVA after exercise have also been observed in Thoroughbred horses. The present study was similar to this previous study; in that, it used echocardiography to confirm right-to-left shunting. However, the mixed solution injected into the vein was succinylated gelatin in the previous study instead of normal saline mixed with room air (15). Echocardiography was performed in a standing position during the bubble study in a previous study on horses. In contrast, the raccoon dogs were placed in a lateral recumbent position in the present study. The IPAVA study involving rats used microspheres from living subjects and isolated lungs, similar to previous studies involving dogs; however, this study attempted to confirm the induction of IPAVA via hypoxia rather than exercise (10). These previous studies have suggested the requirement for continued research on IPAVA in humans and animal species. However, most studies have focused on IPAVA induced by abnormal conditions, such as exercise and hypoxia, and have not focused on IPAVA under normal resting conditions. Furthermore, whether supplemental oxygen lowers Q_IPAVA_ to the same extent in animals as that observed in humans has not been studied. IPAVA continues to be of clinical importance, and human studies have been conducted on the prevalence of IPAVA at rest rather than in specific conditions, such as hypoxia and exercise (19). Differences in various situations and detection methods for vascular injection substances related to IPAVA have been reported. It may be beneficial to compare IPAVA between animal species if a standardised method for IPAVA is continuously applied to various animal species in the future.

### 4.4 Oxygenation and the reduction in Q_*IPAVA*_

Q_IPAVA_ is altered by the concentration of oxygen inhaled. Q_IPAVA_ may increase under hypoxic conditions and decrease under hyperoxic condition (7–12, 45). However, the modulation of IPAVA in animals by high oxygen supplementation remains underexplored. A reduction in BS and the number of bubbles in the LV was observed in all 15 IPAVA-positive raccoon dogs in the present study following supplementation with 100% oxygen via a mask at a rate of 5 L/min for 5 min. Similar findings have been reported in previous human studies (7, 11). Supplementation with 100% oxygen for 5 min was considered sufficient to change Q_IPAVA_ in the present study (11, 18). The statistical results also indicated that oxygenation was effective in reducing the BS and maximum number of bubbles in the LV.

It is unclear how hyperoxia achieved through oxygen supplementation reduces Q_IPAVA_. It has been speculated that IPAVA behaviour is opposite to that of the pulmonary vessels and is more similar to that of the systemic vessels. Thus, it exhibits vasoconstriction during hyperoxia and vasodilation during hypoxia. This may be attributed to IPAVAs being a part of normal foetal circulation, similar to ductus arteriosus (11, 37). Identification of a clear cause for the decrease in Q_IPAVA_ in response to hyperoxia was not possible in the present study. However, as this phenomenon was also observed in animals other than humans, further studies focusing on animal IPAVA, such as comparisons of physiological changes before and after supplemental oxygen, may aid in understanding the phenomenon in humans.

The findings of the present study suggest that Q_IPAVA_ in raccoon dogs was effectively reduced after oxygen supplementation, similar to that observed in humans. Thus, adequate oxygenation effectively reduces blood flow bypassing the pulmonary capillaries. Moreover, it can also prevent pulmonary gas exchange impairment and paradoxical embolism, which are potential clinical manifestations of IPAVA (2, 8, 13, 26, 29, 31, 46). Therefore, the importance of oxygen supplementation can be emphasised in patients whose IPAVA is confirmed through bubble study.

### 4.5 Correlations of IPAVA with cardiac indicators

A higher BS indicates an increase in Q_IPAVA_. The present study confirmed the relationship between BS and several cardiac indicators.

The first is the relationship between IPAVA and pulmonary artery systolic pressure (PASP). TR, which is proportional to PASP, was low or not measured using CW Doppler in most raccoon dogs with IPAVA in the present study. Q_IPAVA_ increases proportionally with PASP through exercise or drug infusion (12, 16). Furthermore, the presence of higher Q_IPAVA_ has been associated with a lower prevalence of PH. Thus, it can be inferred that Q_IPAVA_ increases in response to acutely elevated PASP and relieves PASP to some extent, suggesting that the presence of IPAVA may ameliorate or prevent PH (12, 22). This finding is consistent with the low likelihood of current PH observed in all IPAVA-positive raccoon dogs in the present study. However, determining whether the history of previous PH or the elevated PASP in the raccoon dogs was transient due to excitement during the bubble study is difficult. Furthermore, clear TR was not observed in the IPAVA-negative raccoon dogs; however, the number was small. Therefore, the relationship between IPAVA and PH remains ambiguous and requires further examination.

Q_IPAVA_ may be related to cardiac output rather than to PASP. Increased Q_IPAVA_ has been associated with increased cardiac output with or without increased PASP following the administration of epinephrine in combination with atropine (18). Beta-receptor-expected mediate dilation of IPAVA, which indicates a direct effect on the smooth muscles in the IPAVA, was reported to be an unlikely phenomenon in a previous study. Thus, IPAVA is passively opened by increased cardiac output (16). The relationship between BS and cardiac indicators in healthy raccoon dogs was examined using echocardiography in the present study. The cardiac output per BW was found to be significantly correlated with BS, but not with cardiac output, heart rate, stroke volume, or cardiac output index (cardiac output per BSA). This may be attributed to the BWs of raccoon dogs varying widely in the present study, with the BWs of some raccoon dogs being more than 10-fold higher (BW, 0.5–5.2 kg). Hence, standardisation based on body type rather than the absolute amount of cardiac output would be more beneficial. The sample size of the present study was small. Future studies must consider cardiac output per BW as well as the cardiac output index when analysing correlations with cardiac output.

Several previous studies have investigated the direct factors affecting IPAVA; however, uncertainties still remain (16, 18, 45). The number of raccoon dogs included in this study was small, which limited the ability to clarify the correlations with cardiac indicators. Large-scale studies conducted in the future may yield different results. Therefore, further studies involving various animal species, including raccoon dogs, must be conducted in the future to investigate the correlation between IPAVA and cardiac indicators, such as PASP and cardiac output.

### 4.6 IPAVA based on the maximum number of bubbles in the LV

A BS grading (0–5) system capable of counting the number of bubbles in the LV is often used to determine the severity of IPAVA (19, 41). Although this scoring system is qualitative, it is correlated with Q_IPAVA_ and other indicators. Moreover, its efficacy has been verified (47). Nevertheless, the BS0–5 scoring system has not been formally established, and some studies have used different scoring criteria (4, 23, 40). The difference between consecutive grades of the BS0–5 system is not uniform, i.e., the difference between BS1 and BS2 was not equivalent to that between BS2 and BS3. The intra- and inter-observer reliability determined by counting the maximum number of bubbles in the LV was high in the present study, which is another criterion used to grade the severity of Q_IPAVA_. In contrast to the scoring system, this method has facilitated the quantification of Q_IPAVA_ as it involves counting the number of bubbles individually. In addition, the method of counting bubbles was found to be significantly effective in monitoring the changes in Q_IPAVA_ after the administration of oxygen. Only a few cases had a large number of bubbles that may have led to adequately high deviations in counting (only two cases with >50 bubbles); thus, this consistent measurement method is promising for the quantitative assessment of Q_IPAVA_ using ultrasonography alone.

### 4.7 Limitations

This study has certain limitations. First, the sample size of the present study, including the raccoon dogs with cardiac disease, was 19. Although further large-scale studies must be conducted, the sufficiently low *p*-values ( ≤ 0.001) of “change in BS before and after oxygenation” and “ICC of bubble count” appear to be meaningful enough to overcome the small number of subjects. Furthermore, the detailed medical history and age of the raccoon dogs could not be determined as these were wild animals. Age can affect the prevalence of IPAVA (12, 20). Moreover, additional upright or standing tests or the measurement of the catecholamine concentrations in relation to posture and excitement/strain could not be performed in the present study. This may have influenced the prevalence of IPAVA (16, 19). Following the result of the reduced Q_IPAVA_ of the raccoon dogs in this study, further study of raccoon dogs required for increasing IPAVA such as hypoxia, exercise, and catecholamine injection identified in previous studies of human or other animal species (9, 12, 13, 15, 16). A direct measurement of PASP, as opposed to an indirect measurement using TR, would have yielded more accurate results regarding its correlation with IPAVA. Similarly, arterial blood gas analysis would have provided further insights into the factors affecting IPAVA. The findings of the present study may differ from those of other bubble studies as the BS may differ depending on the microbubble creation protocol (34, 42). Although bubble studies were conducted to determine the presence of assess Q_IPAVA_ and perform a qualitative analysis, microspheres or 99mTC-MAA would have been suitable for the quantitative assessment and determination of the extent of shunting (1, 8, 13). However, microspheres are not yet applicable to live animals and 99mTC-MAA requires additional advanced equipment (1). Thus, bubble studies were conducted owing to the low cost, convenience, and safety.

## 5 Conclusion

The prevalence of IPAVA (75%) was observed in healthy raccoon dogs at rest, suggesting that the prevalence of IPAVA may vary across animal species. Adequate oxygen supplementation was effective in reducing the Q_IPAVA_, which may help prevent potential negative factors such as pulmonary gas exchange impairments and paradoxical embolism that can occur with IPAVA.

## Data availability statement

The original contributions presented in the study are included in the article/supplementary material, further inquiries can be directed to the corresponding author.

## Ethics statement

The animal study was approved by Institutional Animal Care and Use Committee of the Jeonbuk National University. The study was conducted in accordance with the local legislation and institutional requirements.

## Author contributions

CL: Conceptualisation, Data curation, Formal analysis, Investigation, Methodology, Project administration, Resources, Software, Validation, Visualisation, Writing – original draught, Writing – review & editing. MK: Conceptualisation, Data curation, Resources, Writing – original draught, Formal analysis, Writing – review & editing. J-IH: Conceptualisation, Data curation, Resources, Supervision, Writing – original draught, Formal analysis, Writing – review & editing. KL: Conceptualisation, Data curation, Formal analysis, Investigation, Project administration, Supervision, Validation, Visualisation, Writing – original draught, Writing – review & editing. HY: Conceptualisation, Data curation, Formal analysis, Investigation, Methodology, Project administration, Resources, Software, Supervision, Validation, Visualisation, Writing – original draught, Writing – review & editing.
